# Performance of the Attenuation Imaging Technology in the Detection of Liver Steatosis

**DOI:** 10.1002/jum.15512

**Published:** 2020-09-22

**Authors:** Giovanna Ferraioli, Laura Maiocchi, Giovanni Savietto, Carmine Tinelli, Mara Nichetti, Mariangela Rondanelli, Fabrizio Calliada, Lorenzo Preda, Carlo Filice

**Affiliations:** ^1^ Department of Clinical, Surgical, Diagnostic, and Pediatric Sciences University of Pavia Pavia Italy; ^2^ Department of Public Health University of Pavia Pavia Italy; ^3^ Department of Clinical Sciences and Infectious Diseases Fondazione Istituto di Ricovero e Cura a Carattere Scientifico Policlinico San Matteo Pavia Italy; ^4^ Department of Radiology Fondazione Istituto di Ricovero e Cura a Carattere Scientifico Policlinico San Matteo Pavia Italy; ^5^ Department of Clinical Epidemiology and Biometric Unit Fondazione Istituto di Ricovero e Cura a Carattere Scientifico Policlinico San Matteo Pavia Italy; ^6^ Department of Applied Health Sciences Azienda di Servizi Alla Persona di Pavia Pavia Italy; ^7^ Istituto di Ricovero e Cura a Carattere Scientifico Mondino Foundation Pavia Italy

**Keywords:** controlled attenuation parameter, liver steatosis, magnetic resonance, obesity, proton density fat fraction

## Abstract

**Objectives:**

The main aim was to assess the performance and cutoff value for the detection of liver steatosis (grade S > 0) with the Attenuation Imaging–Penetration (ATI‐Pen) algorithm available on the Aplio i‐series ultrasound systems (Canon Medical Systems, Otawara, Japan). The magnetic resonance imaging–derived proton density fat fraction (MRI‐PDFF) was used as the reference standard. Secondary aims were to compare the results to those obtained with the previous ATI algorithm (Attenuation Imaging–General [ATI‐Gen]) and with the controlled attenuation parameter (CAP) and to generate a regression equation between ATI‐Pen and ATI‐Gen values.

**Methods:**

Consecutive adult patients potentially at risk of liver steatosis were prospectively enrolled. Each patient underwent ultrasound quantification of liver steatosis with ATI‐Pen and ATI‐Gen and a CAP assessment with the FibroScan system (Echosens, Paris, France). The MRI‐PDFF evaluation was performed within a week. The correlations between ATI‐Pen, ATI‐Gen, the CAP, and the MRI‐PDFF were analyzed with the Pearson rank correlation coefficient. The diagnostic performance of ATI‐Pen, ATI‐Gen, and the CAP was assessed with receiver operating characteristic curves and an area under the receiver operating characteristic curve (AUROC) analysis.

**Results:**

Seventy‐two individuals (31 male and 41 female) were enrolled. Correlation coefficients of ATI‐Pen, ATI‐Gen, and the CAP with the MRI‐PDFF were 0.78, 0.83, and 0.58, respectively. The AUROCs of ATI‐Pen, ATI‐Gen, and the CAP for detecting steatosis (S > 0) were 0.90 (95% confidence interval, 0.81–0.96), 0.92 (0.82–0.98), and 0.85 (0.74–0.92), and the cutoffs were greater than 0.69 dB/cm/MHz, greater than 0.62 dB/cm/MHz, and greater than 273 dB/m. The regression equation between ATI‐Pen and ATI‐Gen was ATI‐Pen = 0.88 ATI‐Gen + 0.13.

**Conclusions:**

Attenuation Imaging is a reliable tool for detecting liver steatosis, showing an excellent correlation with the MRI‐PDFF and high performance with AUROCs of 0.90 or higher.

## Abstract 2

Access the CME test here and search by article title.

**Abbreviations**
ATI: Attenuation Imaging
ATI‐Gen: Attenuation Imaging–General
ATI‐Pen: Attenuation Imaging–Penetration
AUROC: area under the receiver operating characteristic curve
CAP: controlled attenuation parameter
IQR: interquartile range
MRI‐PDFF: magnetic resonance imaging–derived proton density fat fraction
NAFLD: nonalcoholic fatty liver disease
US: ultrasound

The prevalence of nonalcoholic fatty liver disease (NAFLD) is around 25% in the general population, with an increased risk in patients with obesity, type 2 diabetes, hypertension, or dyslipidemia.^1^, ^2^ A subset of patients with NAFLD will develop nonalcoholic steatohepatitis that may progress to liver cirrhosis with its complications.^2^

Recently, it has been shown that substantial steatosis is associated with fibrosis progression in patients with NAFLD.^3^ Moreover, an association between the quantity of liver fat and the risk of cardiovascular disease in patients with NAFLD has been reported.^4^

Unlike liver fibrosis, liver steatosis is a dynamic process that may improve with appropriate lifestyle changes within a few weeks.^5^, ^6^ Therefore, the possibility of noninvasively detecting the fat in the liver is of great interest. B‐mode ultrasound (US), which is widely used for this purpose, has sensitivity of 60.9% to 65% for the detection of mild steatosis (fat content >5%).^7^, ^8^ Moreover, significant intraobserver and interobserver variability in evaluating the US findings of liver steatosis has been reported.^9^, ^10^

Liver steatosis causes an increased attenuation of the US beam as it traverses the liver parenchyma. By using proprietary algorithms, this attenuation can be objectively quantified, and an attenuation coefficient is calculated.^11^ The controlled attenuation parameter (CAP), implemented in the FibroScan device (Echosens, Paris, France), was the first available tool for this purpose.^12^ Software also based on the quantitation of the attenuation of the US beam has recently been implemented in the Aplio i‐series US systems (Attenuation Imaging [ATI]; Canon Medical Systems, Otawara, Japan). Attenuation Imaging quantifies the attenuation of the US beam in a large area using a real‐time, color‐coded map, automatically filtering out vessels or strong artifacts. By using the magnetic resonance imaging‐derived proton density fat fraction (MRI‐PDFF) as a reference standard, our group has recently shown that the attenuation coefficient calculated with ATI is very promising for the noninvasive assessment of steatosis, performing better than the CAP, with a statistically significant improvement for steatosis grades higher than S1.^13^ In this study, we used the ATI package tuned on a US frequency of 4.0 MHz. To allow better penetration in difficult cases, the manufacturer has lowered the US frequency in the ATI package to 3.0 MHz and has made it a default choice. However, a change in the US frequency used to calculate the attenuation coefficient may give a different result in the same patient.

The main aim of this study was to assess the performance and cutoff value for the detection of liver steatosis (S > 0) of ATI‐Penetration (ATI‐Pen) by using the MRI‐PDFF as the reference standard. Secondary aims were to compare the results to those obtained with the previous ATI software (Attenuation Imaging–General [ATI‐Gen]) and with the CAP and to generate a regression equation between ATI‐Pen and ATI‐Gen values.

## Materials and Methods

This was a cross‐sectional study. From December 2018 to February 2020, consecutive adult patients potentially at risk of liver steatosis, ie, overweight or obese patients and patients with metabolic syndrome, diabetes, or both who complied with the study protocol, were prospectively enrolled. All patients had an alcohol intake of less than 20 g/d. The study protocol was approved by the Ethics Committee of the Fondazione Istituto di Ricovero e Cura a Carattere Scientifico Policlinico San Matteo, and it conformed to the ethical guidelines of the 1975 Declaration of Helsinki. Informed written consent was obtained from all participants. Exclusion criteria were a history of substantial alcohol intake within 2 years of recruitment, secondary causes of liver steatosis, viral or autoimmune hepatitis, infection with human immunodeficiency virus, and genetic disorders.

All patients underwent a clinical evaluation, an anthropometric examination, and fasting biochemical tests. Each patient underwent US quantification of liver steatosis with ATI‐Pen and ATI‐Gen and a CAP and stiffness assessment with the FibroScan system. All studies were performed on the same day by 2 expert operators (G.F. and L.M.), who were blinded to the biochemical data and to MRI‐PDFF results. The MRI‐PDFF evaluation was performed within a week.

Each patient was studied by a single operator who first performed the US quantification of liver steatosis with ATI‐Pen and ATI‐Gen and thereafter the fat and stiffness assessment with the FibroScan device. The MRI‐PDFF evaluation was performed at the radiology unit of the Fondazione Istituto di Ricovero e Cura a Carattere Scientifico Policlinico San Matteo by an expert radiologist (G.S.), who was blinded to biochemical data and to the other imaging results.

Examinations of both ATI and the CAP were performed after a fast of at least 6 hours. The acquisitions were obtained in the right lobe of the liver, through intercostal spaces and with the patient lying in the supine position.

The measurements of the attenuation coefficient with ATI‐Pen and ATI‐Gen were performed with an Aplio i800 US system using a 1–8‐MHz curvilinear transducer. The attenuation coefficient is measured at fixed frequencies (3.0 MHz for ATI‐Pen and 4.0 MHz for ATI‐Gen). The degree of the attenuation of the US signal is color coded in a large field of view. The software automatically removes the influence of the gain and of the US beam profiles. Vessels or strong artifacts are automatically filtered out as well. Attenuation Imaging calculates the attenuation coefficient in decibels per centimeter per megahertz within a large measurement box.

**Figure 1:**
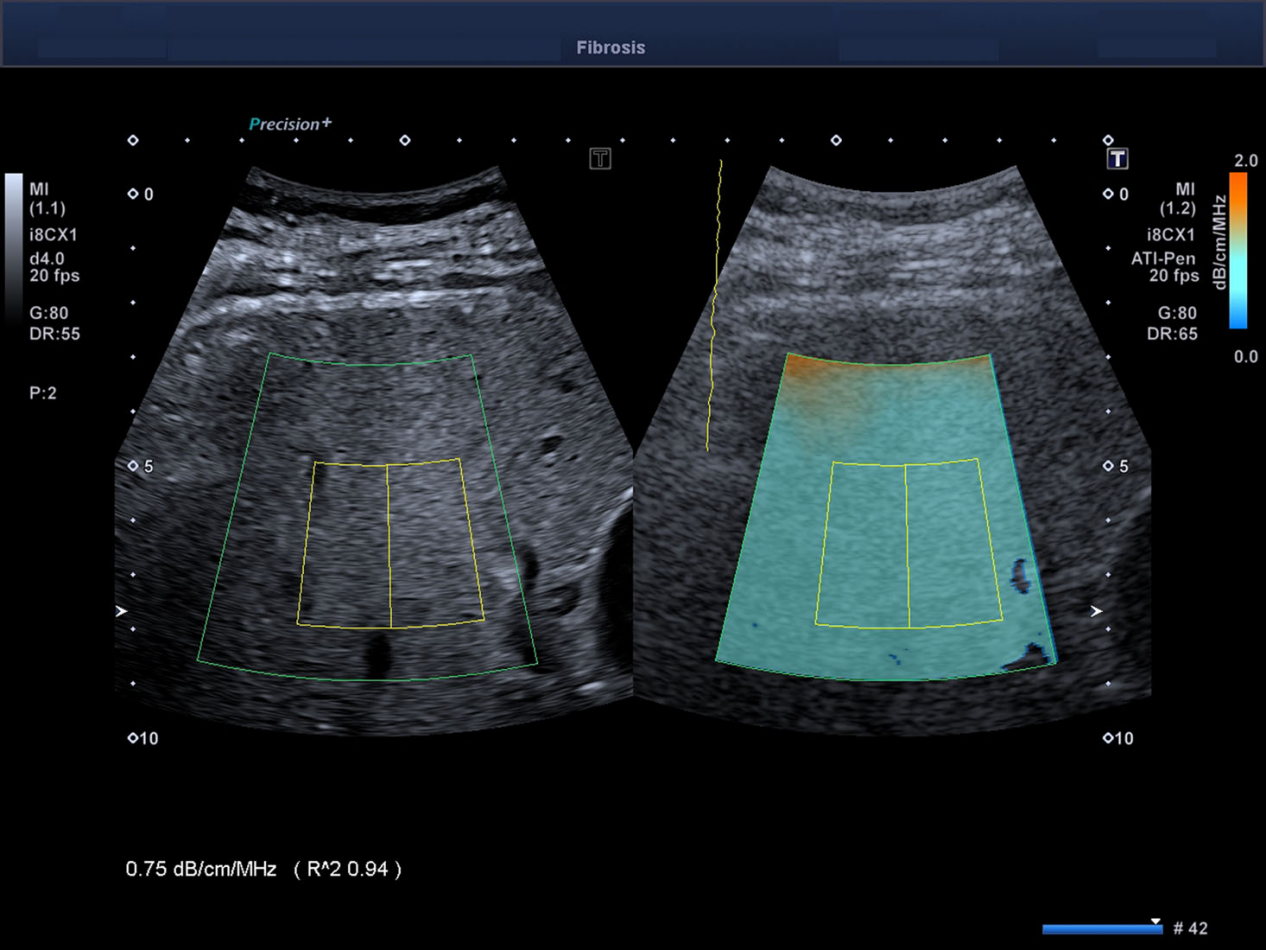
Estimation of the attenuation coefficient with the ATI‐Pen technology in a 55‐year old man with NAFLD. The setting of the color scale is 0.0 to 2.0 dB/cm/MHz. The *R* ^2^ value is 0.94; therefore, the measurement is reliable.

The quality of the measurement is displayed as an *R* ^2^ value; we recorded only the measurements with an *R* ^2^ of 0.90 or higher, as recommended by the manufacturer. The color scale was set 0 to 2.0 dB/cm/MHz (Figure 1). We considered a measurement to have failed when the color‐coded signal was not homogeneously distributed, and there were only sparce spots of color in more than half of the field of view. The median value of 5 consecutive measurements, together with the interquartile range (IQR), was used for the statistical analysis.

The CAP was obtained by using the FibroScan 502 Touch system. A 3.5‐MHz M transducer was used when the skin‐to‐liver capsule distance, estimated with US, was 25 mm or less; otherwise, a 2.5‐MHz XL transducer was used. The FibroScan system estimates both liver stiffness in kilopascals and the liver attenuation coefficient in decibels per meter. The FibroScan system computes the CAP only when the associated liver stiffness measurement is valid, and it uses the same signal as the one used to measure the liver stiffness. As recommended, only liver stiffness measurements with 10 validated measurements and an IQR/median of less than 30% were considered reliable.^14^ There are no recommendations for successful CAP measurement.

The MRI‐PDFF was obtained with a 1.5‐T system (Magnetom Aera; Siemens Healthineers, Erlangen, Germany) using an 18‐channel surface coil in combination with a 32‐channel coil. The procedure that was followed has been described elsewhere.^13^ The detection of liver steatosis (S > 0) was defined by an MRI‐PDFF of greater than 5%.^15^

Descriptive statistics were produced for demographic characteristics for this study sample of patients. The Shapiro–Wilk test was used to test the normal distribution of quantitative variables. When quantitative variables were normally distributed, the results were expressed as the mean value and standard deviation; otherwise, the median and IQR (25th–75th percentiles) were reported. Qualitative variables were summarized as counts and percentages. The correlations between ATI‐Pen, ATI‐Gen, the CAP, and the MRI‐PDFF were analyzed with the Pearson rank correlation coefficient, and they were categorized as follows: 0.00 to 0.25, none or slight; 0.25 to 0.50, fair to moderate; 0.50 to 0.75, moderate to good; and 0.75 to 1.00, almost perfect.^16^ The diagnostic performance of ATI or the CAP was assessed with receiver operating characteristic curves and an area under the receiver operating characteristic (AUROC) curve analysis. The optimal cutoff for detecting S of greater than 0 was determined by the Youden index to maximize sensitivity and specificity. The method described by DeLong et al^17^ for correlated data was used for comparing the AUROCs. A regression equation between ATI‐Pen and ATI‐Gen values was obtained by a linear regression analysis. The data analysis was performed with the Stata statistical package (release 15; StataCorp, College Station, TX) and MedCalc statistical software (version 18.11; (MedCalc Software bvba, Ostend, Belgium).

## Results

Seventy‐two individuals (31 male and 41 female; mean age, 52.5 ± 14.9 years; mean body mass index, 30.8 ± 5.0 kg/m^2^) were enrolled in the study. Three individuals did not undergo the MRI‐PDFF evaluation because of claustrophobia. There were 2 failures with ATI‐Pen, 3 failures with ATI‐Gen, and 1 failure with the CAP. The baseline characteristics of the study cohort are summarized in Tables 1 and 2. All ATI values were obtained with an IQR/median of less than 30%.

**Table 1:** Main Clinical and Demographic Characteristics of the Study Cohort

Characteristic	Overall (n = 72)	Male (n = 31)	Female (n = 41)	*P*
Age, y	52.5 ± 14.9	53.5 ± 13.0	51.8 ± 16.3	.63
BMI, kg/m^2^	30.8 ± 5.0	30.4 ± 4.9	31.2 ± 5.2	.52
Waist circumference, cm	105.3 ± 14.3	107.5 ± 12.5	103.6 ± 15.4	.25
Diabetes, n (%)	10 (13.9)	5 (16.1)	5 (12.2)	.74
AST, IU/L	23.2 ± 10.0	26.5 ± 13.0	21.2 ± 7.0	.06
ALT, IU/L	22.5 (18–35)	30 (20–59)	19 (17–29)	.003
Glycemia, mg/dL	100.0 ± 36.4	112.5 ± 56.0	92.6 ± 12.8	.05
Triglycerides, mg/dL	109 (77–149)	136 (80–163)	99 (76–140)	.16
Cholesterol, mg/dL	204 ± 53	189 ± 63	212 ± 46	.13
Platelet count, 10^9^/L	250 ± 64	223 ± 55	271 ± 63	.004
GGT, IU/L	24.5 (16–43)	36.5 (17–48)	19.5 (14–34.5)	.31
Liver stiffness, kPa	5.1 ± 2.3	5.3 ± 1.6	5.0 ± 2.7	.56
MRI‐PDFF, %^a^	6.2 (3.9–14)	9.9 (5.6–17.9)	5.0 (3.1–9.4)	.007
ATI‐Pen, dB/cm/MHz	0.71 ± 0.12	0.77 ± 0.12	0.66 ± 0.1	.0002
ATI‐Gen, dB/cm/MHz	0.66 ± 0.12	0.71 ± 0.11	0.62 ± 0.12	.002
CAP, dB/m	285 ± 51	304 ± 50	271 ± 46	.006

Data are presented as mean ± SD and median (IQR) where applicable. ALT indicates alanine aminotransferase; AST, aspartate aminotransferase; BMI, body mass index; and GGT, γ‐glutamyl transferase.

^a^ The MRI‐PDFF was not obtained in 3 patients because of claustrophobia.

**Table 2:** Main Clinical and Demographic Characteristics of the 69 Patients With MRI‐PDFF Results

Characteristic	Overall (n = 69)	MRI‐PDFF ≤5% (n = 24)	MRI‐PDFF >5% (n = 45)	*P*
Age, y	52.8 ± 14.6	45.8 ± 15.9	56.6 ± 12.5	.003
Female, n (%)	38 (55.1)	20 (83.3)	18 (40)	.0008
BMI, kg/m^2^	30.9 ± 5.1	28.7 ± 4.2	32.1 ± 5.3	.009
Waist circumference, cm	104.5 ± 12.9	95.7 ± 9.2	109.2 ± 12.0	.00001
Diabetes, n (%)	10 (14.5)	1 (4.2)	9 (20)	.15
AST, IU/L	23.3 ± 10.2	22.3 ± 10.5	23.9 ± 10.1	.57
ALT, IU/L	23.5 (18–37)	19 (15–27)	27.5 (19.5–38)	.24
Glycemia, mg/dL	100.5 ± 37.0	87.1 ± 5.6	109.6 ± 45.7	.03
Triglycerides, mg/dL	111 (76–149)	78.5 (67–114)	140 (95–163)	.001
Cholesterol, mg/dL	204.4 ± 54.4	204.3 ± 53.7	204.5 ± 55.6	.99
Platelet count, 10^9^/L	248.6 ± 65.0	262.4 ± 61.3	242.4 ± 66.5	.30
GGT, IU/L	26 (16–43)	16 (12–47)	27 (18–43)	.09
Liver stiffness, kPa	5.1 ± 2.3	4.7 ± 1.4	5.3 ± 2.7	.34
MRI‐PDFF, %	6.2 (3.9–14)	3.4 (2.75–3.95)	10.6 (6.7–17.9)	<10^5^
ATI‐Pen, dB/cm/MHz	0.71 ± 0.12	0.61 ± 0.06	0.77 ± 0.11	<10^5^
ATI‐Gen, dB/cm/MHz	0.66 ± 0.12	0.55 ± 0.05	0.72 ± 0.11	<10^5^
CAP, dB/m	285 ± 51	245 ± 41	307 ± 43	<10^5^

Data are presented as mean ± SD and median (IQR) where applicable. ALT indicates alanine aminotransferase; AST, aspartate aminotransferase; BMI, body mass index; and GGT, γ‐glutamyl transferase.

Liver steatosis was present in 45 of 69 (65.2%) individuals with MRI‐PDFF results available. The patients with liver steatosis were older, had a significantly higher body mass index and waist circumference, and showed significantly higher values of blood glucose and triglycerides.

Table 3 reports the results of the correlation analysis. The correlation coefficients of ATI‐Pen and ATI‐Gen with the MRI‐PDFF were higher than that of the CAP (*r* = 0.78 and 0.83 versus 0.58).

**Table 3:** Correlations Between ATI‐Pen, ATI‐Gen, MRI‐PDFF, and CAP Values Analyzed by the Pearson *r* Correlation

Parameter	ATI‐Pen	CAP	MRI‐PDFF
ATI ‐Pen		0.70	0.78
ATI‐Gen	0.87	0.53	0.83
CAP	0.70		0.58

**Figure 2:**
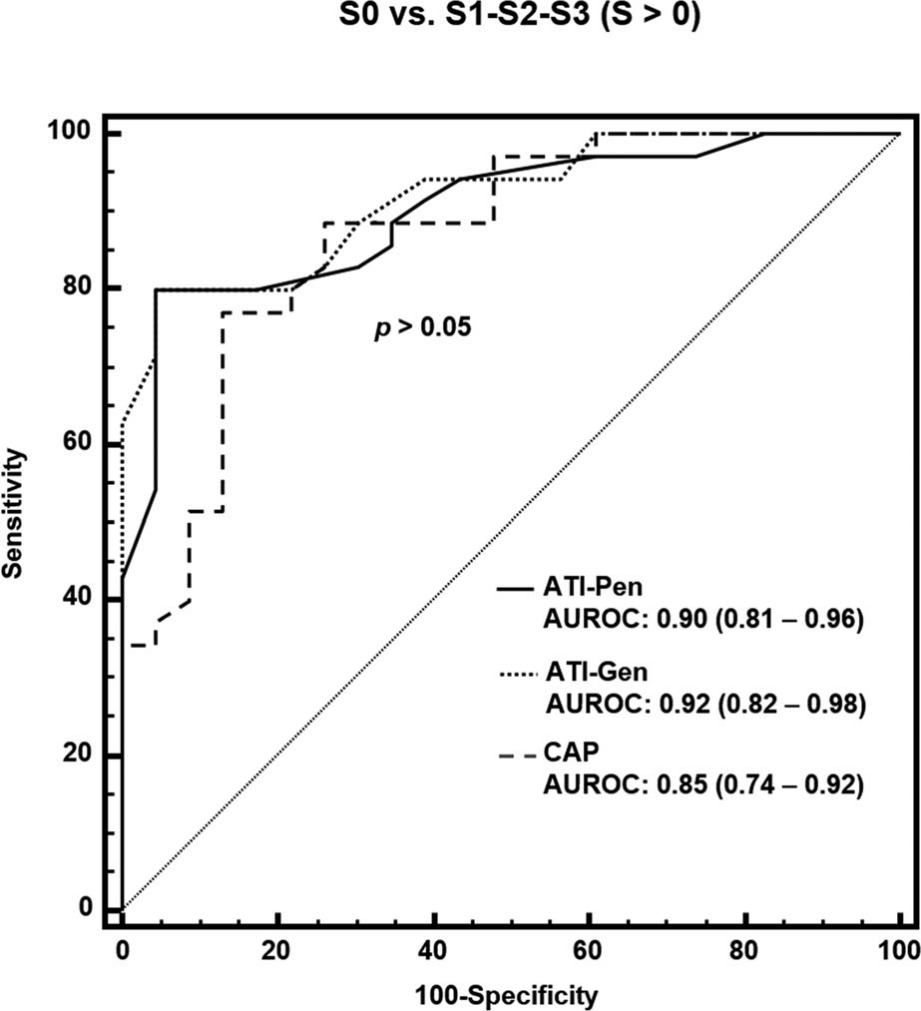
Comparison of receiver operating characteristic curves for ATI‐Pen, ATI‐Gen, and CAP for S0 versus S1 to S3 (S > 0), as defined by MRI‐PDFF of greater than 5%.

The diagnostic performance of ATI‐Pen, ATI‐Gen, and the CAP is reported in Table 4. The AUROCs of ATI‐Pen, ATI‐Gen, and the CAP for detecting steatosis (S > 0) were 0.90 (95% confidence interval, 0.81–0.96), 0.92 (0.82–0.98), and 0.84 (0.72–0.92), respectively, and the cutoffs were greater than 0.69 dB/cm/MHz, greater than 0.62 dB/cm/MHz, and greater than 273 dB/m. The performance of ATI‐Pen and ATI‐Gen was higher than that of the CAP; however, the differences were not statistically significant (Figure 2). The regression equation between ATI‐Pen and ATI‐Gen was ATI‐Pen = 0.88 ATI‐Gen + 0.13.

**Table 4:** Clinical Performance of ATI‐Pen, ATI‐Gen, and CAP Using Optimal Cutoff Values

Parameter	Method	S0 vs S1–S3 (MRI‐PDFF >5%)
Cutoff, dB/cm/MHz Cutoff, dB/cm/MHz Cutoff, dB/m	ATI‐Pen ATI‐Gen CAP	>0.69 >0.62 >273
AUROC	ATI‐Pen ATI‐Gen CAP	0.90 (0.81–0.96) 0.92 (0.82–0.98) 0.85 (0.74–0.92)
Sensitivity, %	ATI‐Pen ATI‐Gen CAP	78.6 (63.2–89.7) 81.1 (64.8–92.0) 80.0 (65.4–90.4)
Specificity, %	ATI‐Pen ATI‐Gen CAP	95.8 (78.9–99.9) 95.6 (78.1–99.9) 83.3 (62.6–95.3)
PPV, %	ATI‐Pen ATI‐Gen CAP	97.1 (84.4–99.9) 96.8 (82.9–99.9) 90.0 (76.1–97.4)
NPV, %	ATI‐Pen ATI‐Gen CAP	71.9 (53.3–86.3) 75.9 (56.1–89.9) 69.0 (48.8–85.0)
LR+	ATI‐Pen ATI‐Gen CAP	18.9 (15.8–22.5) 18.6 (15.6–22.3) 6.4 (3.8–6.0)
LR–	ATI‐Pen ATI‐Gen CAP	0.22 (0.03–1.7) 0.20 (0.03–1.5) 0.24 (0.08–0.7)

Values in parentheses are 95% confidence intervals. LR indicates likelihood ratio; NPV, negative predictive value; and PPV, positive predictive value.

## Discussion

**Figure 3:**
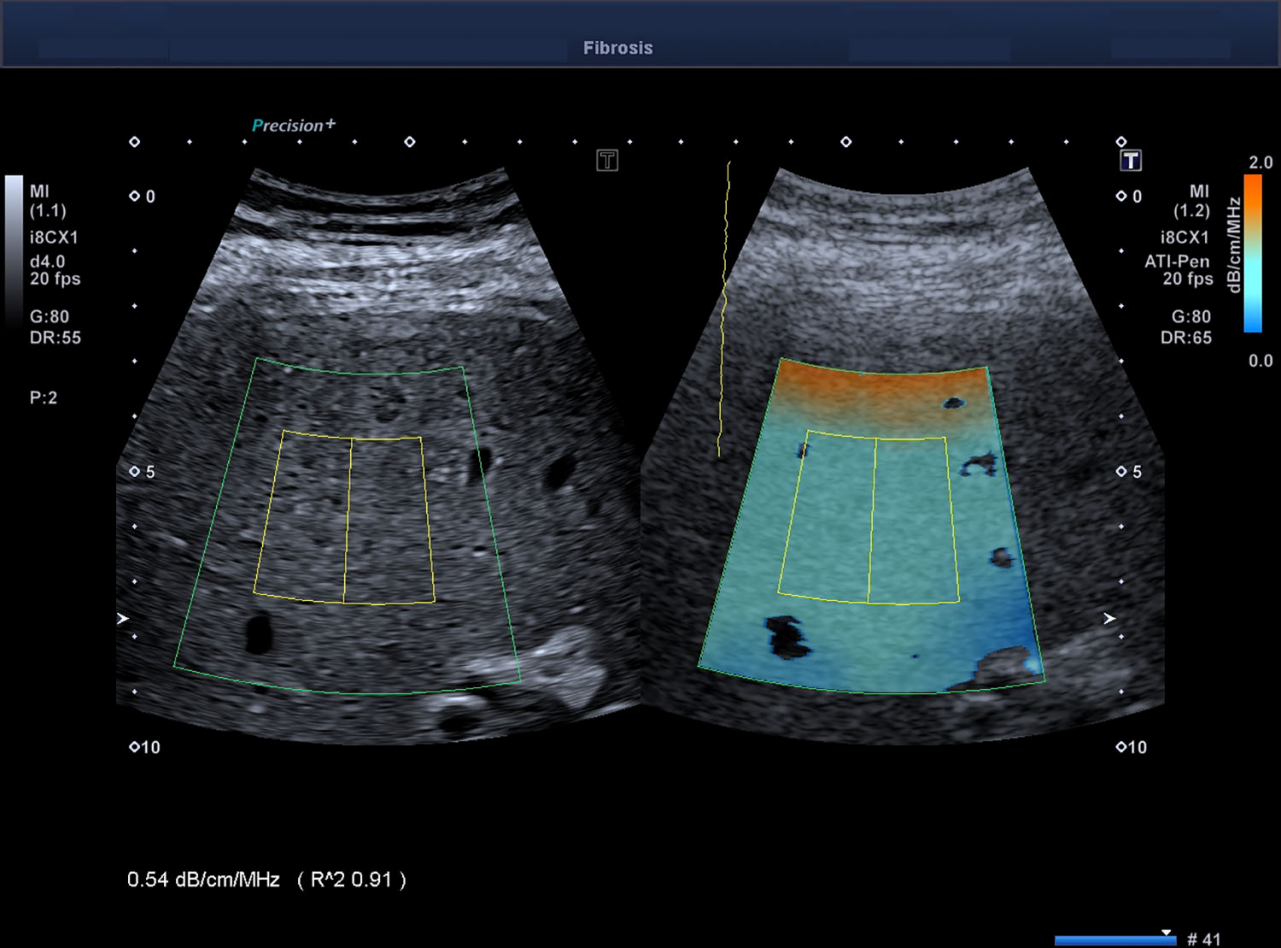
Assessment of liver steatosis with ATI‐Pen. A thick orange line, due to a reverberation artifact, is shown in the near field. In the far field, there are 2 dark‐blue areas, likely due to high noise with a weak echo signal. These areas must not be included in the measurement box: ie, the yellow rectangle inside the region of interest. The *R* ^2^ value is 0.91; therefore, the measurement is reliable.

The results of this study demonstrate that ATI‐Pen and ATI‐Gen show high performance in detecting liver steatosis, with an AUROC of 0.90 or higher for both. The correlation coefficients with the MRI‐PDFF of both ATI‐Pen and ATI‐Gen were higher than that of the CAP (0.78 and 0.83 versus 0.58). These findings agree with that of a recent study published by our group.^13^ The CAP, which is implemented in the FibroScan device, has been available for 10 years, and its assessment has become a point‐of‐care technique for the quantification of liver steatosis. The measurements are standardized, with a region of interest that has a cylindric shape of 10 mm wide and 40 mm long, and measurements are taken between 25 and 65 mm below the skin surface with the M transducer and between 35 and 75 mm with the XL transducer. Moreover, per the manufacturer's recommendation, the transducer is positioned on the skin in a limited area. This protocol allows a standardized measurement because the device does not provide a real‐time B‐mode image of the liver; however, it also limits the assessment to a somewhat specific area. Differently from the CAP, the calculation of the attenuation coefficient with ATI is guided by the B‐mode image, allowing the choice of the best area for the measurement. Moreover, the color‐coded display of the attenuation coefficient helps in detecting areas of artifacts, which may appear as a thick orange line or dark blue areas. The former is generally evident in the near field and is probably due to reverberation artifacts, whereas the latter are usually observed posterior to blood vessels or in the far field, likely due to high noise with a weak echo signal (Figure 3).

Attenuation Imaging is a novel technology, and few studies have been published to date.^13^, ^18^, ^19^, ^20^, ^21^, ^22^, ^23^ Interestingly, in a series of 101 patients scheduled for liver biopsy for mixed etiologies of liver disease, in which ATI‐Pen was used, the same cutoff found in our study for diagnosing any grade of steatosis (S1–S3) was obtained, with sensitivity and specificity of 76% and 86%, respectively.^23^ As in the study by Dioguardi Burgio et al,^23^ we used the ATI quality factor recommended by the manufacturer: ie, an *R* ^2^ value of 0.90 or higher.

The ATI‐Gen algorithm is set at a US frequency of 4.0 MHz, whereas ATI‐Pen is based on a US frequency of 3.0 MHz. We found that the optimal cutoff of ATI‐Gen for the detection of steatosis was 0.62 dB/cm/MHz, similar to that obtained in our previous study,^13^ whereas the optimal cutoff of ATI‐Pen was higher (0.69 dB/cm/MHz). It is well known that the attenuation is directly related to the transducer frequency: ie, the higher the frequency, the higher the attenuation. Therefore, this difference in cutoff values is understandable. On the other hand, the frequency dependency when calculating the US attenuation coefficient should be considered when comparing results obtained with different US systems or with the same system using a different setting. This could be an important source of variability, and an agreement between manufacturers is desirable to mitigate the differences in attenuation coefficient estimates between US systems. The regression equation generated in our study can be used in clinical practice for converting ATI‐Gen values to ATI‐Pen values or vice versa.

This study had some limitations. First, because of the small sample size, the performance of the ATI tool in grading liver steatosis was not assessed. However, since B‐mode US has low sensitivity in the detection of liver steatosis, we believe that the availability of a tool that helps in the diagnosis of liver steatosis in patients at risk is of great interest. Second, liver biopsy was not performed in our series of patients. However, it should be acknowledged that liver biopsy is not the first‐choice procedure for screening purposes. On the other hand, it has been demonstrated that the MRI‐PDFF is highly accurate in the assessment of liver steatosis, and it has become the reference standard in clinical trials.^24^ Third, the regression equation between ATI‐Pen and ATI‐Gen was obtained in a small series of individuals. Further studies in larger cohorts are needed to confirm this finding.

In conclusion, ATI is a reliable tool for the detection of liver steatosis, showing an excellent correlation with the results obtained with the MRI‐PDFF and high performance, with AUROCs of 0.90 or higher.
